# Phenytoin as a novel anti-vitiligo weapon

**DOI:** 10.1186/1740-2557-2-11

**Published:** 2005-11-22

**Authors:** MR Namazi

**Affiliations:** 1 Dermatology Department, Shiraz University of Medical Sciences, Shiraz, Iran

**Keywords:** Dilantin, Treatment, Melanocyte, Immunity, Cytokines

## Abstract

Vitiligo is a psychologically devastating clinical conundrum which affects approximately 1% of the general population. The exact cause of the illness is an enigma, but several hypotheses about its pathogenesis are advanced.

The autoimmune hypothesis proposes an autoimmune attack against melanocytes. Although anti-melanocyte autoantibodies have been demonstrated in vitiligo, recent research casts doubt on their pathogenic role and instead supports the involvement of cell-mediated autoimmune response in the pathobiology of this disorder, which is characterized by increase of suppressor T-cells and decrease of the helper/suppressor ratio in association with the presence of type-1 cytokine secreting cytotoxic T cells in the vicinity of disappearing melanocytes.

The neural hypothesis proposes that increased release of norepinephrine, a melanocytotoxin, from the autonomic nerve endings in the microenvironment of melanocytes injures these cells. Moreover, norepinephrine induces the catecholamine degrading enzyme monoamine oxidase (MAO), which favors the formation of toxic levels of hydrogen peroxide in the vicinity of melanocytes.

Another theory suggests that abnormal permeability of melanosome membrane, which normally prevents the diffusion of toxic melanin precursors into the cytoplasm, may cause melanocyte damage.

Phenytoin, the widely-used anticonvulsant, has been employed both topically and systemically in the treatment of some dermatological disorders. The drug has been shown to significantly suppress mitogen-induced activation of lymphocytes and cytotoxic T lymphocyte activity and to polarize the immune response toward the type-2 pathway. It also significantly decreases suppressor T cells and increases the helper/suppressor ratio.

At high concentrations, the drug inhibits the release of norepinephrine and the activity of MAO. Moreover, phenytoin is suggested to interact with membrane lipids, which may promote stabilization of the membranes.

The hydantoin moiety of phenytoin exerts a direct stimulatory action on melanocytes; facial hyperpigmentation is a recognized side effect of orally administered phenytoin.

Altogether, the above evidence suggests that phenytoin could be therapeutically effective against vitiligo. As phenytoin stimulates collagen production and inhibits its breakdown, its concomitant use with topical steroids could prevent steroid-induced skin atrophy while potentiating the anti-vitiligo effect of these agents.

## 

Vitiligo, a psychologically devastating and frequently recalcitrant skin disorder, affects approximately 1% of the general population [1]. Theories concerning the cause of vitiligo have focused on three different mechanisms: auto-immune, neural, and autocytotoxic. The autoimmune hypothesis proposes an autoimmune attack against melanocytes. The autocytotoxic theory postulates that cytotoxic precursors to melanin synthesis accumulate in melanocytes, causing cell death. The neural hypothesis proposes that the elevated levels of some neurotransmitters and catecholamine degrading enzymes injure melanocytes [2].

The important aspects of each theory pertinent to the present paper are discussed below.

## I) Evidence for involvement of cell-mediated immunity in the pathogenesis of vitiligo

Although melanocyte-specific autoantibodies have been demonstrated in vitiligo, their pathogenic role remains uncertain [1]. Though these autoantibodies have been shown to be able to damage pigment cells both in vitro and in vivo, more recently it has been reported that specific autoantibodies for tyrosinase are present at low frequency in the sera of vitiligo patients, and that such autoantibody titers may be found both in vitiligo-like depigmentation (melanoma-associated depigmentation) and in healthy controls [3]. The patchy distribution of cutaneous depigmentation and the most frequent symmetrical distribution of lesions corroborate the concept that autoimmune melanocyte damage is caused by clones of lymphocytes with affinities for specific areas of skin [1]. In fact, high frequencies of circulating melanocyte-specific CD8+ T cells expressing skin-homing capacity and exerting anti-melanocyte cytotoxicity in vitro have been found in vitiligo patients [3]. Moreover infiltrating activated T lymphocytes have been observed at the periphery of vitiligo lesions and a recent immunopathologic study of lesional skin of vitiligo patients noted a high frequency of cutaneous lymphocyte antigen-positive activated cytotoxic T cells clustered in perilesional skin adjacent to the disappearing melanocytes [4]. An increased level of soluble interleukin (IL)-2 receptor, IL-6 and IL-8 has been observed in vitiligo patients, which further suggests that T cell activation may be a component in vitiligo pathogenesis [5].

Notably, perilesional T-cell clones exhibited a predominant type-1-like cytokine secretion profile, whereas the degree of type-1 deviation in uninvolved skin-derived T-cell clones correlated with the process of microscopically observed melanocyte destruction in situ. Detailed analysis of broad spectrum of cytokines produced by lesional- and nonlesional-derived CD4+ and CD8+ T-cell clones confirmed deviation toward type-1-like in both CD4 and CD8 compartments, which mirrored depigmentation process observed locally in the skin [4]. The facts that vitiligo is found more frequently in patients with metastatic melanoma and is associated with an improved prognosis, and that vitiligo-like depigmentation has been observed following immunotherapy of melanoma, further support a crucial role for cell-mediated immunity in the pathogenesis of the disorder [5].

It deserves noting that the peripheral blood of patients with vitiligo shows a significant decrease in helper cells and helper/suppressor ratios in comparison with control subjects [6].

An association between CD8+ T lymphocyte reactivity to the melanocyte antigen gp100, and to a lesser extent Melan A/MART-1, and vitiligo has been demonstrated. Furthermore, the disease activity appears correlated with reactivity to gp100 [5].

## II) Involvement of increased norepinephrine (NE) and monoamine oxidase (MAO) levels in the pathogenesis of vitiligo

It has recently been demonstrated that axon terminals and epidermal melanocytes make contact via chemical synapses in human skin [2,7].

An increased release of catecholamine from the autonomic nerve endings in the microenvironment of melanocytes in the vitiliginous areas has been reported. NE is known for having direct and indirect toxic effects on melanocytes [2,7].

Direct actions include interacting with cellular sulfhydryl groups, enzyme inhibition, impairing mitochondrial calcium uptake, and forming some cytotoxic products such as free radicals. Indirect actions include activating α-receptors of skin arterioles causing a severe vasoconstriction, thereby producing toxic oxygen radicals due to hypoxia [2,7]. Moreover, elevated level of NE secreted by keratinocytes or by nerve endings induces the catecholamine degrading enzyme monoamine oxidase (MAO). Increased MAO activity favors the formation of toxic levels of hydrogen peroxide, which is not buffered due to the low catalase activity in vitiliginous skin [2,7].

## III) Abnormal permeability of melanosome membrane as a potential pathogenic factor in vitiligo

Tyrosine, a derivative of phenol, is oxidized into melanin via a complex series of oxidative reactions, often accompanied by electronic rearrangements within molecules. Some of these products are unstable and capable of forming radicals that react with other molecules in the cell. It is thought that melanin synthesis is confined within the melanosome to prevent these melanin precursors from diffusing into the cell where they might disrupt essential metabolic pathways, leading to the destruction of the pigment cell.

Defects either inherited or acquired in the membranes of melanosomes resulting in the demise of melanocytes are supposed to cause vitiligo [8].

## IV) Phenytoin exerts an inhibitory effect on the pathomechanism behind vitiligo but a direct stimulatory action on melanocytes

Phenytoin (dilantin), a diphenyl-substituted hydantoin, is a widely prescribed anticonvulsant agent which has been used in cutaneous medicine in the treatment of ulcers, inflammatory conditions, and epidermolysis bullosa [9]. Interestingly, this agent possesses a myriad of effects which make it potentially effective against vitiligo:

Phenytoin is documented to significantly suppress mitogen-induced activation of lymphocytes [10,11], cytotoxic T lymphocyte activity [11-14] and cell-mediated immunity [15], polarizing the immune response toward a type-2-like cytokine secretion profile [14]. Additionally, it significantly decreases suppressor T-cells and increases the helper/suppressor ratio [16,17].

Phenytoin inhibits the production of superoxide anion by immune cells [18] and significantly improves rheumatoid arthritis, a cell-mediated autoimmune disorder [19].

Interestingly, some *in vitro *studies have shown that this drug, at high concentrations, which may also be reached by the topical application, inhibits the release of NE and inhibits the activity of MAO [20]. it is also speculated that the agent may interact with membrane lipids (and conceivably with melanosome membrane lipids as well), which could promote the stabilization of the membranes [20].

Melasma (chloasma), the facial hypermelanosis produced by hyperfunctional melanocytes, is a recognized side effect of orally administered phenytoin [9,21]. It has been suggested that the hydantoin moiety exerts a direct action on the melanocytes; it has been shown to expand melanophores in amphibian larvae [21].

In conclusion, given its inhibitory effect on cell-mediated immunity, NE release, and MAO activity and also in view of its potential ability to stabilize melanosome membrane and to stimulate melanocytes, phenytoin may prove to be effective against vitiligo. Importantly, as phenytoin facilitates collagen deposition and inhibits collagenase activity [9], its concomitant topical use with steroids may prevent steroid-induced skin atrophy while potentiating the anti-vitiligo effect of these agents. The latter effect could occur through phenytoin's ability to directly stimulate melanocyte proliferation, which is lacked by steroids. Moreover, the immunomodulatory effect of this agent, which may be employed topically in relatively high concentrations without producing the untoward systemic effects, may permit the reduction of the concentration of the topical steroids (steroid-sparing effect) and thus their frequently-occurring dermatologic side effects. As the closing comment, topically-administered phenytoin was shown to exert significant immunomodulatory activity and be effective against lichen planus [22], which could reasonably be the case in vitiligo as well.

## V) Testing this hypothesis

This hypothesis can be tested using animal models of vitiligo such as the Smyth chicken. The Smyth chicken expresses the major features of human vitiligo – the delayed development of cutaneous amelanosis, most commonly appearing during adolescence or at young adulthood, and variable in occurrence, location and extent [23]. The etiology of vitiligo in the Smyth chicken appears to include an inherent defect in the melanocytes and an associated autoimmune response [24]. Though the Smyth chicken does not incorporate all the genetic and environmental factors which may do or cause vitiligo in humans, it is an excellent model for the study of human vitiligo in that it parallels the human condition in variability, complexity, mode of expression, and polyfactorial etiology. As complement to animal studies, this hypothesis could be tested most rigorously in patients suffering from bilateral lesions by applying a topical preparation of phenytoin to the lesion(s) of one side of the body and a topical placebo to the contralateral lesion(s), then comparing the results (double-blind, placebo-controlled, bilateral paired comparison method).
